# 

**Published:** 1858-10

**Authors:** 


### EDITORIAL.

### TO SUBSCRIBERS.

According to the notice we gave in the July number, this is the last copy of the Journal that will be sent to any subscriber who is indebted for more than the present current volume. We had hoped that three or four months' notice would have induced most of those on the delinquent list to pay up, but many have not done so, and we certainly shall not furnish them with the Journal longer gratuitously. We shall continue, however, to print a large number of extra copies, and any who may send in the amount of their indebtedness after the issue of this number, can have their volumes completed. None need delay sending the amount of their subscriptions through fear of its being lost in the post-office, for we assume all the risk; and in every instance where we are assured that money has been properly mailed, we credit it to the individual, whether it has ever reached us or not. If any who have sent the amount of their subscriptions find their Journal stopped, let them inform us at once, and we will correct the error.

### RECEIPTS FOR JOURNAL UP TO NOVEMBER 6th, 1858.

### Illinois.

| | | |
|---|---|---|
| Dr. F. B. Haller, | vol. 1, 1858, | $2 |
| " L. Hale, | " " | 2 |
| " J. B Meigs, | " " | 2 |
| " A. Fisher, | " " | 2 |
| " N. P. Holden, | " " | 2 |
| " G. Schlœtzer, | " " | 2 |
| " A. P. Finch, | " " | 2 |
| " J. T. Pearman, | " " | 2 |
| " J. F. Spilman, | " " | 2 |
| " A. S. Haskell, | " " | 2 |
| " J. W. Morgan, | " " | 2 |
| " Wm H. Studley, | vol. 6 & 1, 1857-8, | 4 |
| " L. L. Lake, | " " " | 4 |
| " B. Harris, | vol. 5, 6 & 1, 1856-7-8, | 6 |
| " A. H. Luce, | vol. 1 & 2, 1858-9, | 4 |

### Michigan.

| | | |
|---|---|---|
| Dr. B. S. Hawley, | vol. 1, 1858, | $2 |
| " W. Cargin, | vol. 2, 1859, | 2 |
| " H. Lockwood, | vol. 4, 5 & 6, 1855-6-7, | 5 |

### Indiana.

| | | |
|---|---|---|
| Dr. John Lewis, | vol. 1, 1858, | $2 |
| " J. D. Gray, | " " | 2 |
| " John Pickard, | " " | 2 |
| " J. H. Harriman, | " " | 2 |
| " R. A. Perkins, | vol. 6 & 1, 1857-8, | 4 |
| " A. Beelers, | vol. 4, 5, 6, 1. 1855-6-7-8, | 8 |
| " Geo. Fletcher, | vol. 5 & 6, 1856-7, | 4 |

### Ohio.

| | | |
|---|---|---|
| Dr. Lamb, | vol. 5, 6 & 1, 1856-7-8, | $6 |

### Kentucky.

| | | |
|---|---|---|
| Dr. O. H. Seeds, | vol. 1, 1858, | $2 |

### Iowa.

| | | |
|---|---|---|
| Dr. D. A. Kittle, | vol. 1, 1858. | $2 |
| " H. L. Whitman, | vol. 4,5,6,1, 1855-6-7-8, | 8 |
| " W. A. Daniels, | vol. 6, 1 & 2, 1857-8-9, | 6 |

